# The Waterfall Fascia Lata Interposition Arthroplasty “Grika Technique” as Treatment of Posttraumatic Osteoarthritis of the Elbow in a High-Demand Adult Patient: Validity and Reliability

**DOI:** 10.1155/2018/8253732

**Published:** 2018-04-22

**Authors:** Giuseppe Rollo, Roberto Rotini, Denise Eygendaal, Paolo Pichierri, Ante Prkic, Michele Bisaccia, Riccardo Maria Lanzetti, Domenico Lupariello, Luigi Meccariello

**Affiliations:** ^1^Department of Orthopedics and Traumatology, Vito Fazzi Hospital, Lecce, Italy; ^2^Shoulder and Elbow Unit, Rizzoli Orthopedic Institute, Bologna, Italy; ^3^Upper Limb Unit, Department of Orthopedic Surgery, Amphia Hospital, Breda, Netherlands; ^4^Department of Orthopedic Surgery, AMC, Amsterdam, Netherlands; ^5^Orthopedics and Traumatology Unit, SM Misericordia Hospital, University of Perugia, Perugia, Italy; ^6^Orthopedics and Traumatology Unit, Univerisity of Rome La Sapienza, Rome, Italy

## Abstract

**Introduction:**

The elbow interposition arthroplasty is a very common procedure performed mainly on active young patients who need great functionality and for whom total joint replacement is contraindicated and arthrodesis is noncompliant. We are going to demonstrate a case of a 34-year-old male suffering from malunion of the distal humerus, elbow stiffness, and manifest signs of arthrosis of the dominant limb, treated with the IA Grika technique at a 5-year follow-up.

**Patients and Methods:**

The chosen criteria to evaluate the injured side and the uninjured side during the clinical and radiological follow-up were the objective function and related quality of life, measured by the Mayo Elbow Performance Score (MEPS), and postoperative complications. To assess flexion and supination forces and elbow muscular strength, a hydraulic dynamometer was used.

**Results:**

At a 5-year follow-up, the results were excellent as during the first year.

**Conclusions:**

The Grika technique is a valid and feasible option in the treatment of elbow injuries.

## 1. Introduction

Management of elbow arthritis in young patients poses a dilemma in treatment options. Elbow arthritis is a debilitating condition producing pain, stiffness, and functional loss [1]. Etiology varies from the rare primary elbow osteoarthrosis, to more common rheumatoid arthritis and posttraumatic osteoarthritis [1]. Surgery for osteoarthritis includes arthroscopic debridement, resection arthroplasty, interposition arthroplasty, ulnohumeral prosthesis, total elbow prosthesis, and arthrodesis [2]. The purpose of this case report is to describe and demonstrate how the Grika technique proved an excellent salvage option in the posttraumatic elbow osteoarthrosis in a young adult at a 5-year follow-up.

## 2. The Grika Surgical Technique

**Figure 1:**
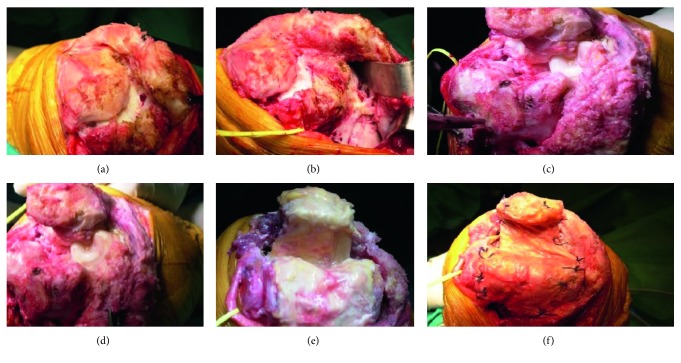
Peroperative situation. Posterior arthrotomy (a); marking and preservation of the ulnar nerve (yellow loop) (b); debridement of the ulnohumeral joint (c, d); good cartilage quality on the radial head (d); debrided ulnohumeral joint (e); interposition arthroplasty with sutured fascia lata graft, like a waterfall (f).

The surgical technique is based on our previous elbow trauma surgery experiences. We used a longitudinal posterior incision over the old scar and blunt dissection with careful hemostasis to approach the elbow joint (Figures 1(a) and 1(b)). First of all, the ulnar nerve was identified and left in situ without transposition (Figures 1(b) and 1(c)). Then we identified and opened the Kocher interval between the anconeus and the carpi ulnaris muscles. Both the lateral and medial collateral ligaments were released from the humerus. A release of the distal tendon of the brachial triceps was performed for complete overview of the elbow joint (Figures 1(c)–1(e)).

Arthrolysis and total synovectomy were performed to maximize range of motion (Figures 1(c)–1(e)) the head of the radius was assessed to have sufficient cartilage quality (Figure 1(d)). The allogeneic fascia lata was interposed like a waterfall from the olecranon over the coronoid process up to the posterior side of the humeral articular surface. Using no. 2 Vicryl (Johnson & Johnson, New Brunswick, NJ, USA), two transosseous Krackow locking stitches at the olecranon and humerus were placed along cascade sutures on all edges of the graft (Figure 1(f)).

After suturing the graft, we reduced the elbow and reinserted the medial and lateral collateral ligaments, strengthened with allograft iliotibial band, were sutured with two Krackow locking stitches using no. 2 Ethibond (Johnson & Johnson, New Brunswick, NJ, USA) into their physiological position. The tendon of the brachial triceps was reanchored with four Krackow locking stitches using no. 2 Ethibond (Johnson & Johnson, New Brunswick, NJ, USA).

**Figure 2:**
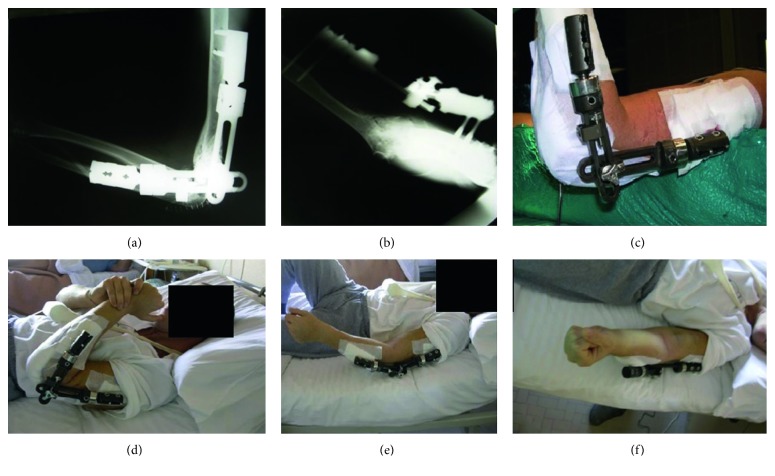
Postoperative situation. Postoperative radiographs after Grika interposition arthroplasty with hinged external fixator (a, b); active and passive motion during hospitalization (c–f).

After performing stability tests of the elbow and assessing the range of motion, we applied a hinged external elbow fixator (Orthofix, Verona, Italy), with 4 mm of extra distraction (Figure 2).

## 3. Case Presentation

**Figure 3:**
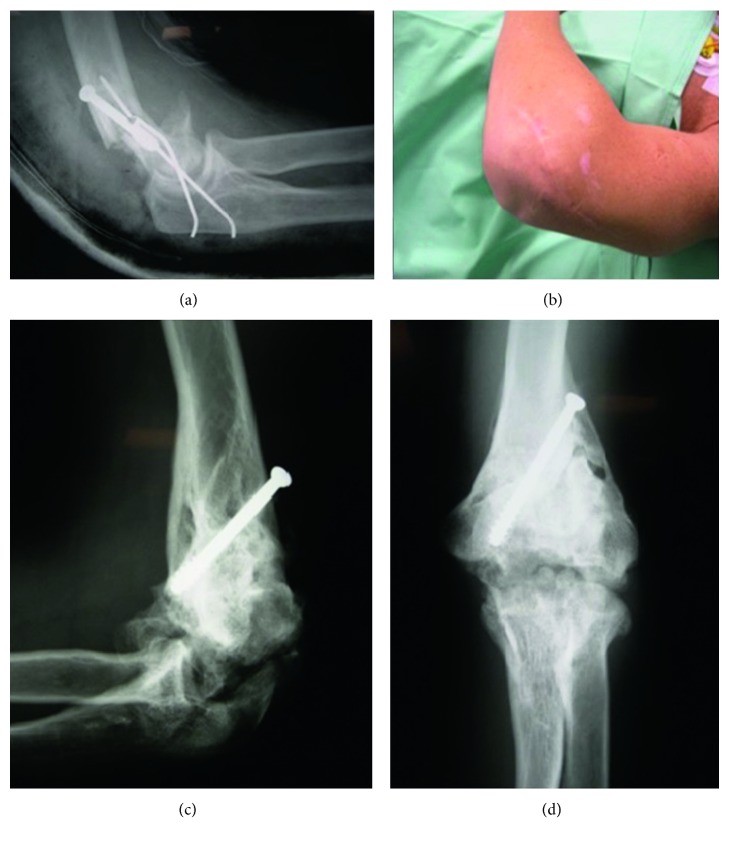
Direct postoperative radiograph showing the insufficient fracture fixation after previous surgery (a); the skin showing the scar following previous surgery (b); radiographs showing distal humeral malunion and generalized elbow joint osteoarthritis (c, d).

**Figure 4:**
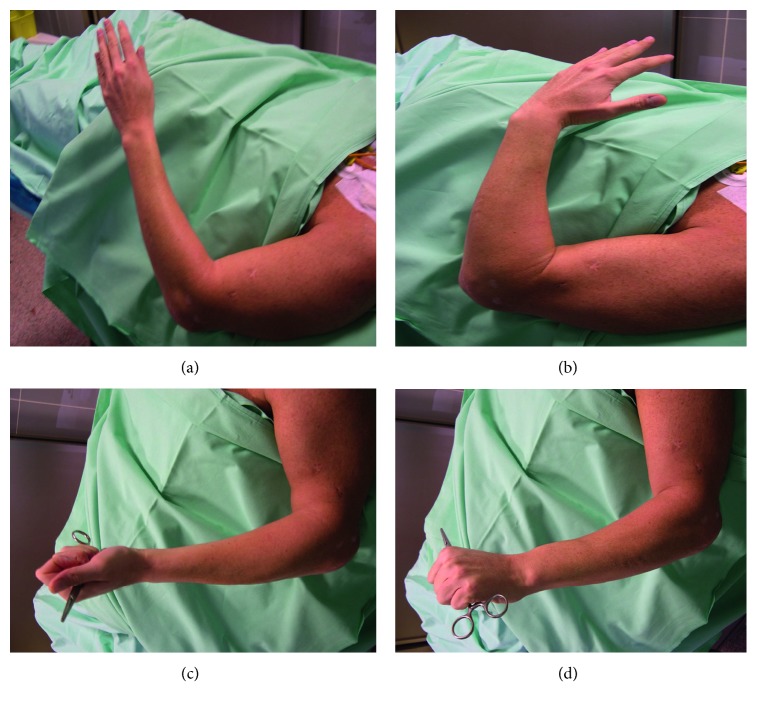
Preoperative situation and active elbow range of motion. Maximum of 80 degrees of flexion (a); no possibility of extension (b); sufficient pronation and supination (c, d).

A 34-year-old male patient was admitted to our center. He had malunion of the distal humerus of his dominant arm (Figure 3), accompanied with elbow stiffness (Figure 4), and evident signs of osteoarthritis were seen on radiographs (Figures 3(c) and 3(d)).

The study was approved by the hospital's Ethical Review Board, and it was conducted in accordance with the principles of the Declaration of Helsinki and its amendments. We fully informed the subject, and he gave his consent.

The patient underwent Grika interposition arthroplasty (see surgical technique paragraph and Figure 1) followed by a suitable rehabilitation protocol (see Rehabilitation Protocol and Figure 2). The external fixator which was used for initial support was removed 14 weeks after surgery.

The injured side (IS) and the noninjured side (NIS) were compared during the clinical follow-up. Function was evaluated with subjective quality of life measured by the Mayo Elbow Performance Score (MEPS) [3].

Objective function was evaluated by range of motion, flexion strength, and supination strength. A calibrated hydraulic dynamometer was used for the strength measurements. During these strength measurements, five measurements were taken by the same evaluator. The mean score of the last four was calculated as the first measurement was disregarded to avoid bias because of a learning curve caused by the patient's awareness.

**Figure 5:**
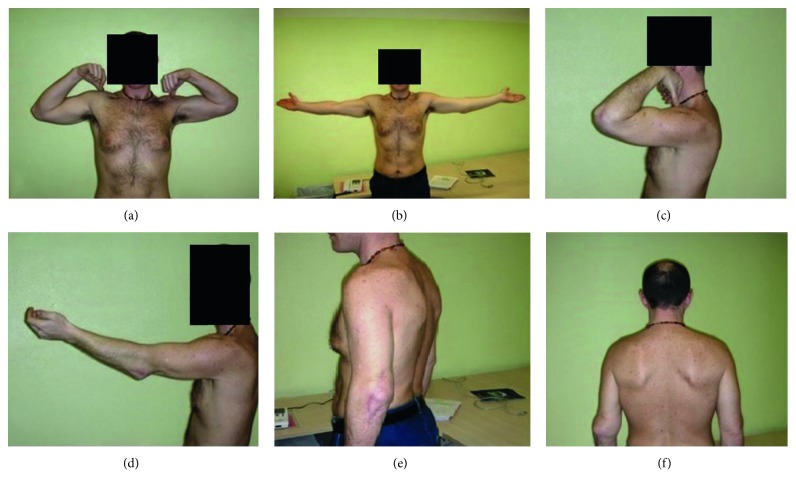
Final postoperateive situation at 5 years of follow-up (a–d). Valgus axial deviation (e, f).

At 1 year after surgery, both the MEPS and muscle strengths showed a difference in favor of the NIS. At 5 years of follow-up, the IS had a flexion strength of 98% of the NIS (28.9 N versus 29.5 N) and supination strength of 98% of that in the NIS (4.6 versus 4.7) and a MEPS of 100. Functional outcomes are shown in Figure 5.

The patient was monitored for any postoperative complications.

## 4. Rehabilitation Protocol

### 4.1. Postoperative Recovery

*Assisted supination and pronation with elbow in 90 degrees of flexion with the arm horizontally. Shoulder range of motion as needed based on evaluation of the physiotherapist, avoiding excessive anteflexion.*

Week 1: active, pain-free flexion and extension combined with assisted passive motions.

 
*Range of motion exercises*: Active and assisted passive elbow flexion from 45 degrees of flexion to full flexion and supination with the arm horizontally.

### 4.2. Strengthening Programme: Part 1

*The patient wears a protective brace, except during rehabilitation. Single-plane flexion-extension is trained.*

Week 2: submaximal pain-free biceps isometric contractions with elbow in 90 degrees of flexion.

Week 3: single-plane active pain-free elbow flexion, extension, supination, and pronation.

Week 4: pain-free active flexion; 30 degrees of flexion to full elbow flexion with HEEF.

Week 5: pain-free active flexion; 20 degrees of flexion to full elbow flexion with HEEF.

Week 6: pain-free active flexion; 10 degrees of flexion to full elbow flexion with HEEF.

 
*Range of motion exercises*: active ROM elbow flexion and extension, pain-free.

### 4.3. Strengthening Programme: Part 2

*Continuing pain-free single plane active elbow flexion, extension, supination, and pronation.*

Week 7–11: full range of motion of HEEF and elbow; discontinue brace if adequate motor control without brace.

 
*Range of motion exercises*: continue active ROM elbow flexion and extension, pain-free. May begin composite motions, that is, extension with pronation.

If at 8 weeks postoperatively the patient has significant range of motion deficits, physiotherapist may consider more aggressive management, after consultation with referring surgeon, to regain range of motion.

### 4.4. Strengthening Programme: Part 3

*Elbow flexion, extension, supination, and pronation against resistance are progressively allowed.*

Week 12: removal of HEEF.

After week 12: may start light upper extremity weight training. Initiate endurance program that simulates desired work activities and requirements.

## 5. Discussion

The purpose of interposition arthroplasty is to effectively reduce pain and improve functionality in young patients with elbow osteoarthritis without compromising future surgical options. Sears et al. suggested this requires an accurate evaluation of the compliance and the functional demands of the patients [2].

Ulnohumeral arthroplasty is performed for mild and moderate degeneration and may be carried out arthroscopically or open with good functional outcomes. However, the elbow may be at risk of intra-articular fractures immediately after surgery, and a certain caution is required before resuming sports activities [4]. Total elbow arthroplasty is performed in patients with osteoarthritis, yet according to literature, it seems to be less favorable and with a greater risk of complications in younger and more demanding patients [5]. Resection arthroplasty and arthrodesis are not feasible for the young and demanding patient, as the consequent loss of function is highly disabling and therefore should be performed only as a last resort [1].

The interposition arthroplasty is one of the oldest reconstructive options for elbow arthritis and other joints, described for a variety of disorders [6]. For years, different elbow interposition tissues have been utilized, varying from synthetic grafts to Achilles tendon allografts [2], free rectus abdominis muscle flaps [7], scapular flaps [2, 6], and the anconeus muscle [6, 8, 9], with or without the addition of a hinged external fixator [1, 10, 11]. This procedure is considered as a salvage option in patients for whom conservative treatment failed, and total elbow arthroplasty is contraindicated [12].

In this specific case, our decision to return to the past, to the Vittorio Putti technique, is based on discoveries that interposition grafts microscopically form a zone with endothelial lined sacs [6, 13]. Melvin Henderson already in 1918 reported that the outcomes of interposition arthroplasty were better in the temporomandibular joint (93% of good outcomes), compared to the elbow (78%), the hip (57%), and the knee (15%) [6]. The high rate of failures in the lower limbs was probably caused by weight bearing [13]. In reexamining the scientific articles published by Vittorio Putti, it is interesting to notice his emphasis placed on the use of the fascia lata [6]. The preference of fascia lata originates from the composition of the fascia lata, as it is rich in collagen. The high collagen content seems to be a rational choice for grafting into osteoarthritic joints. The flap of the fascia lata, used by Putti and in our technique, has a considerable similarity with the small intestinal submucosa and decellularized dermis [1, 14, 15].

In contrast to the original Putti technique, we have used a cadaveric fascia lata allograft instead of an autologous graft. Using an autologous graft would result in donor site morbidity and allografts were not available in his time [16]. Another difference between our technique and that of Putti is that our cascade suturing not only fixates the interposition but also ensures covering of the entire joint. Besides, the cascade effect allows neovascularization of the fascia lata through the arterial vessels from the anterior capsule, which are the main protagonists of blood supply to this articular zone [17].

Furthermore, we have used iliotibial allografts to reconstruct the medial and lateral collateral ligaments. Trauma-surgical experience on the ankle and foot demonstrates that iliotibial band allografts showed excellent outcomes in 92% of the cases [18]. Additionally, Lindenhovius and Jupiter [19] describes surgically unblocking the rigid elbow usually necessitates the release of the posterior fascia of the medial collateral ligament, debridement of all calcifications and anterioposterior capsulotomy.

We have used a hinged elbow external fixation to protect the grafts from high-impact loads, as the use of the hinged elbow external fixator had good functional outcomes and good subjective results after extensive releases of the stiff elbow [11, 20]. Besides, distraction itself might have a positive influence on regeneration of the affected tissues [21–23].

## 6. Conclusion

This case report is to show the first 5-year result of our interposition arthroplasty technique. It also emphasizes that an old surgical technique can be a good solution when a surgical problem is presented which does not fit into standard care programs. This is comparable to the Grika language dialect of the senior author, which is a dialect in the south of Italy dating back to the ancient Greeks but still in use and actual.
